# Blog and Podcast Watch: Neurologic Emergencies

**DOI:** 10.5811/westjem.2016.9.31010

**Published:** 2016-10-25

**Authors:** Andrew Grock, Nikita Joshi, Anand Swaminathan, Salim Rezaie, Chris Gaafary, Michelle Lin

**Affiliations:** * Olive View, UCLA Medical Center, Department of Emergency Medicine, Sylmar, California; † Stanford University, Department of Emergency Medicine, Stanford, California; ‡ New York University, Bellevue Hospital Center, Department of Emergency Medicine, New York, New York; § University of Texas Health Science Center, Department of Emergency Medicine, San Antonio, Texas; ¶ University of Tennessee Health Science Center, Department of Emergency Medicine, Memphis, Tennessee; || University of California, San Francisco, Department of Emergency Medicine, San Francisco, California; # Keck School of Medicine of the University of Southern California and the Los Angeles County + USC Medical Center, Department of Emergency Medicine, Los Angeles, California

## Abstract

**Introduction:**

The *WestJEM* Blog and Podcast Watch presents high quality open-access educational blogs and podcasts in emergency medicine (EM) based on the ongoing ALiEM Approved Instructional Resources (AIR) and AIR-Professional series. Both series critically appraise resources using an objective scoring rubric. This installment of the Blog and Podcast Watch highlights the topic of neurologic emergencies from the AIR series.

**Methods:**

The AIR series is a continuously building curriculum that follows the Council of Emergency Medicine Residency Director’s (CORD) annual testing schedule. For each module, relevant content is collected from the top 50 Social Media Index sites published within the previous 12 months, and scored by eight board members using five equally weighted measurement outcomes: Best Evidence in Emergency Medicine (BEEM) score, accuracy, educational utility, evidence based, and references. Resources scoring ≥30 out of 35 available points receive an AIR label. Resources scoring 27–29 receive an honorable mention label, if the executive board agrees that the post is accurate and educationally valuable.

**Results:**

A total of 125 blog posts and podcasts were evaluated. Key educational pearls from the 14 AIR posts are summarized, and the 20 honorable mentions are listed.

**Conclusion:**

The *WestJEM* Blog and Podcast Watch series is based on the AIR and AIR-Pro series, which attempts to identify high quality educational content on open-access blogs and podcasts. This series provides an expert-based, post-publication curation of educational social media content for EM clinicians with this installment focusing on neurologic emergencies.

## BACKGROUND

Despite the rapid rise of social media educational content available through blogs and podcasts in emergency medicine (EM),^1^ identification of quality resources for educators and learners has only received preliminary progress.^2^–^4^ In 2008, the Accreditation Council for Graduate Medical Education endorsed a decrease in synchronous conference experiences for EM residency programs by up to 20% in exchange for asynchronous learning termed Individualized Interactive Instruction (III).^5^ Residency programs, however, are often unsure how to identify quality online resources specifically for asynchronous learning and III credit.

To address this need, the Academic Life in Emergency Medicine (ALiEM) Approved Instructional Resources (AIR) Series and AIR-Pro Series were created in 2014 and 2015, respectively, to help EM residency programs identify quality online content specifically on social media.^6^,^7^ Using an expert-based, crowd-sourced approach, these two programs identify trustworthy, high-quality, educational blog and podcast content. This *WestJEM* Blog and Podcast Watch series presents annotated summaries written by the editorial Board from the AIR and AIR-Pro Series.

This installment from the AIR Series summarizes the highest scoring social media educational resources on neurologic emergencies.

## METHODS

### Topic Identification

The AIR series is a continuously building curriculum based on the CORD testing schedule (http://www.cordtests.org/).

### Inclusion and Exclusion Criteria

A search of the 50 most frequently visited sites per the Social Media Index^9^ was conducted for resources relevant to neurologic emergencies, published within the previous 12 months. The search, conducted in December 2015, included blog posts and podcasts, and those written in English were included for our scoring by our expert panel.

### Scoring

Extracted posts were scored by eight reviewers from the AIR Editorial Board, which is comprised of EM core faculty from various U.S. medical institutions. The scoring instrument contains five measurement outcomes using seven-point Likert scales: Best Evidence in Emergency Medicine (BEEM) score, accuracy, educational utility, evidence based, and references (Table 1).^8^ More detailed methods are described in the original description of the AIR series.^7^ Board members with any role in the production of a reviewed resource recused him/herself from grading that resource.

### Data Analysis

Resources with a mean evaluator score of ≥ 30 points (out of a maximum of 35) are awarded the AIR label. Resources with a mean score of 27–29 and deemed accurate and educationally valuable by the reviewers are given the honorable mention label.

## RESULTS

We initially included a total of 125 blog posts and podcasts. We describe key educational pearls from the 14 AIR posts and list the 20 honorable mentions (Table 2).

### AIR Content

#### 1. Simon E. Reversal of Anticoagulation in a True Emergency. EM Docs. (November 10, 2015). http://www.emdocs.net/reversal-of-anticoagulation/

This blog post reviews anticoagulants such as vitamin K antagonists, director thrombin inhibitors (DTIs), and factor 10a inhibitors as well as their mechanism of action, pharmacokinetics, reversal agents, and management strategies.

##### Take-Home Points

Vitamin K antagonists, such as warfarin, can be reversed by vitamin K, fresh frozen plasma (FFP), and prothrombin complex concentrate (PCC). FFP infusions can be limited by the rate of infusion and the large volume required, in comparison with PCC which has neither of these limitations. PCC is indicated to urgently reverse warfarin in a major hemorrhagic event.

DTIs, such as dabigatran, block free thrombin and clot-bound thrombin and lack specific reversal agents. [Editorial note - since this blog publication, an antibody reversal agent, idarucizumab, has been made available]. Hemodialysis can clear approximately 35% of this drug, and PCC has a potential role in reversal, although it lacks significant evidence at this point. Also lacking evidence at this time is the recommendation by the American College of Cardiology Foundation and the American Heart Association (AHA) for transfusion of packed red blood cells and FFP to reverse hemorrhagic events while on DTIs. For reversal of Factor 10a inhibitors, such as rivaroxaban, apixaban, and fondaparinux, PCC shows promise, and a specific reversal agent is reportedly in development.

#### 2. Long, B. Controversies in the Diagnosis of Subarachnoid Hemorrhage. EM Docs. (November 20, 2015). http://www.emdocs.net/controversies-in-the-diagnosis-of-subarachnoid-hemorrhage/

The most recent guidelines by the American College of Emergency Physicians and the AHA recommend a non-contrast head computed tomography (CT) followed by lumbar puncture as the gold standard for diagnosing a subarachnoid hemorrhage (SAH). Recent advances have changed the diagnostic approach to SAH. This post reviews the strengths and limitations of different diagnostic approaches including these three clinical decision rules: CT followed by lumbar puncture (LP), CT alone if performed in less than six hours from headache onset, and CT angiography.

##### Take-Home Points

The Ottawa SAH clinical decision tool approaches 100% for ruling out SAH, but has poor specificity and currently lacks external validation. According to the most current literature, the risk of SAH is less than 1% after a negative non-contrast head CT performed within six hours of headache onset as interpreted by a neuroradiologist. CT angiography performed after a non-diagnostic CT may increase the sensitivity of ruling out SAH and may be reasonable for patients where LP is not feasible. After a negative non-contrast head CT, cerebrospinal fluid (CSF) xanthochromia can also be used to diagnose a SAH, but it can take 2–12 hours to develop. Thus, xanthrochromia may be absent if the LP is performed less than 12 hours after headache onset. Differentiating between a traumatic LP and a SAH can be difficult and no externally validated studies exist to support a specific cut-off. As there are diagnostic problems with each of the possible SAH work-ups, CT alone, CT+CT angiography, CT+LP, shared decision-making should be applied.

#### 3. George W, Kulkarni M. Endovascular Stroke Therapy: Is this the New Standard? EM Docs. (September 8, 2015). http://www.emdocs.net/endovascular-stroke-therapy-is-this-the-new-standard/

This blog post reviews the most recent literature regarding endovascular treatment for acute ischemic stroke. The results, limitations, and responses to each of the following studies are discussed: MR CLEAN, EXTEND-IA, ESCAPE, SWIFT PRIME, and REVASCAT.^10^^–14^ The blog authors acknowledge that these studies seem promising for improving stroke outcomes but heed caution that providers should be wary of its use outside of selected study populations.

##### Take-Home Points

The most recent studies regarding endovascular therapy for acute ischemic stroke (MR CLEAN, EXTEND-IA, ESCAPE) show improved outcomes compared to tissue plasminogen activator (tPA) alone in the select population studied. Earlier studies (MERCI, SYNTHESIS, MR RESCUE) failed to show true benefit of endovascular intervention. The AHA and the American Stroke Association have endorsed endovascular therapy in their most recent guidelines by stating that there is clinical benefit only in patients with large vessel occlusions and salvageable brain tissue.

#### 4. Rezaie, SR. Minor Head Trauma in Anticoagulated Patients: Admit for Observation or Discharge? Rebel EM. (July 20, 2015). http://rebelem.com/minor-head-trauma-in-anticoagulated-patients-admit-for-observation-or-discharge/

This blog reviews the controversial disposition for head trauma patients on warfarin or clopidogrel after an initial negative head CT due to the concern for delayed intracranial hemorrhage. The author critically appraises the 2012 prospective observational multicenter study on traumatic intracranial hemorrhages in patients with pre-injury warfarin and clopidogrel use.^15^

##### Take-Home Points

Routine head CTs in head-injured patients with current warfarin or clopidogrel use should be performed, even in well-appearing patients. As delayed traumatic intracranial hemorrhage in head-injured patients on therapeutic warfarin is rare, this post reports he/she may be discharged home after an initial negative head CT. They require, however, clear discharge instructions and close follow up. No patients on clopidogrel had a delayed intracranial hemorrhage. Though no firm evidence-based medicine recommendations exist, patients may require 24-hour hospital observation if they have any of the following: difficulty accessing emergent medical care secondary to poor functional capacity, long travel times, or no friend/family member to observe them should they medically deteriorate. Patients with supratherapeutic anticoagulation, blunt head trauma, and a negative initial head CT were not explicitly discussed in the literature reviewed, but this post recommends a low threshold to admit for frequent neurological checks, repeat INR (international normalized ratio) measurements while holding anticoagulation, and possibly a repeat head CT if any neurologic decline develops.

#### 5. Chan, T. ALiEM-Annals of EM Journal Club: Clinical Decision Rule for Subarachnoid Hemorrhage. Academic Life in Emergency Medicine. (January 20, 2014). http://www.aliem.com/journal-club-clinical-decision-rule-subarachnoid-hemorrhage/

This blog features a live Google Hangout with Dr. Jeff Perry and Dr. Ian Stiell, the lead authors of “Clinical decision rules to rule out subarachnoid hemorrhage for acute headache” published in JAMA 2013.^16^ Clinical decision rules discussed by the paper were outlined. Topics discussed by the authors include the following: How a patient’s location in the emergency department (ED) may bias his/her workup; the approach to counseling patients about the role of the LP for ruling out SAH; and the value of a radiology resident’s interpretation of CT in ruling out SAH.

##### Take-Home Points

For headache patients, providers must avoid framing bias and not let the patient care location (i.e. fast track area) influence the work up. For SAH, shared decision-making with patients should be used after a negative head CT obtained within six hours of headache onset, because the SAH rate is extremely low. In significantly anemic patients, however, blood may appear isodense on CT and increase the likelihood of a falsely negative interpretation. Although inexperienced CT interpreters may miss a small SAH, there were no major adverse outcomes in the study’s cohort.

#### 6. Crucco, A. SGEM#112: Bang Your Head – Paediatric Concussions. Skeptics Guide to Emergency Medicine (March 22, 2015). http://thesgem.com/2015/03/sgem112-bang-your-head-paediatric-concussions/

This blog review includes two studies on the management of pediatric concussions and relates each to a clinical case. Two clinical questions are addressed: Is there benefit to recommending strict rest after a child has a concussion?, and is there benefit to using intravenous hypertonic saline as a therapy for pediatric concussive pain? The author evaluates each study with the blog’s 11-point “Quality Checklist for Randomized Clinical Trials.”

##### Take-Home Points

In children with concussions, two days of strict rest, as defined by no school, work, or physical activity, followed by a gradual return to activity is preferred over five days of rest followed by a gradual return to activity. Hypertonic (3%) saline should not be used for treatment of moderate to severe concussion in pediatric patients until higher quality studies support its use.

#### 7. Spyres, M. Swaminathan A. Seizure, “Answers”. EM Lyceum (December 9, 2014). http://emlyceum.com/2014/12/09/seizure-answers/

This thoroughly referenced resource discusses ED patients with seizures, specifically regarding first- and second-line medications, decision for neuroimaging, and diagnosis of pseudo-seizures.

##### Take-Home Points

Based on the current best available evidence, intravenous (IV) lorazepam, intramuscular (IM) midazolam, and per rectum (PR) diazepam are equally reasonable first-line medications for seizures depending on the route available. Patients with first-time seizures do not universally require neuroimaging in the ED, but those with an abnormal mental status, focal neurologic deficits, trauma, immunocompromised status, or focal seizures should prompt emergent imaging. Pseudo-seizures (or psychogenic nonepileptic seizures) have a number of distinctive features that help to differentiate them from true seizures, including prolonged duration, pelvic thrusting, side-to-side head movements, and absence of postictal confusion.

#### 8. Kreitzer, N. Swaminathan A. Spinal Cord Injury “Answers”. EM Lyceum (April 14, 2015). http://emlyceum.com/2015/04/14/spinal-cord-injury-answers/

This well-referenced blog review focuses on various topics related to spinal cord injuries, including optimal imaging modality, management of compression fractures, cervical spine clearance after a negative CT of the cervical spine, and treatment of neurogenic shock.

##### Take-Home Points

CT imaging is superior to plain films of the spine particularly in regard to the assessment of potential cervical spine injuries. There is limited evidence to guide management of neurogenic shock but using norepinephrine as a first-line medication appears reasonable. Elderly patients with compression fractures and an absence of neurologic symptoms can be discharged home if they are able to ambulate and safely perform their daily activities of living. There are multiple options for cervical spine clearance after a negative cervical spine CT including urgent magnetic resonance imaging (MRI), immobilization and follow up, and immobilization with delayed flexion-extension films. [Editorial note: Early evidence suggests that there is little additional value in obtaining flexion-extension films after a negative CT in neurologically intact, awake, adult patients.^17,18^]

#### 9. Swaminathan A, Junck E. SGEM#106: O Canada-Canadian CT Head Rule for Patients with Minor Head Injury. Skeptics Guide to Emergency Medicine. (February 3, 2015). http://thesgem.com/2015/02/sgem106-o-canada-canadian-ct-head-rule-for-patients-with-minor-head-injury/

This 34-minute podcast and blog post summarizes the two minor head injury decision instruments (New Orleans and Canadian). The review begins with a case and then reviews each decision tool. The authors then discuss more in depth the studies the tools were derived from, and compare and contrast these instruments.

##### Take-Home Points

Both the Canadian and New Orleans head CT decision tools are highly sensitive for positive CT findings and clinically important brain injuries. The Canadian CT Head Tool had higher specificity and may be more clinically applicable as it is designed to predict clinically significant brain injuries.

#### 10. Spampinato N. Post Lumbar Puncture Headaches. Rebel EM. (March 31, 2015). http://rebelem.com/post-lumbar-puncture-headaches/

This blog post reviews the prevention and treatment of post-LP headaches. Evidence-based techniques and preventative measures are reviewed to help minimize this disabling complication.

##### Take-Home Points

Post-LP headache prevention techniques include the following: using smaller 20–22 gauge spinal needles, positioning the needle bevel parallel to the dural fibers, replacing the stylet before withdrawal of the spinal needle, and minimizing the number of LP attempts. Per the evidence reviewed, post-LP headaches are not affected by bed rest, the volume of cerebrospinal fluid removed, the patient position, and IV fluids prior to the LP. Finally, IV and oral caffeine do seem to improve post-LP headaches, but these headaches have a high recurrence rate.

#### 11. Huang, D. PEM Pearls: Migraine Treatment for Pediatric EM Patients. Academic Life in Emergency Medicine. (August 31, 2015). http://www.aliem.com/migraine-treatment-for-pediatric-em-patients/

This blog review provides an in-depth analysis of the evidence for pediatric headaches and migraine therapies. This topic is important for clinicians, because three-fourths of pediatric patients diagnosed with primary headaches are diagnosed with migraines.

##### Take-Home Points

For pediatric migraines, the preferred medication is prochlorperazine for children older than six years. Compared to metoclopramide, prochlorperazine decreases repeat visits as well as the need for rescue medications, admission rate, disposition time, and hypotensive events compared to chlorpromazine. Diphenhydramine can be used to reduce akathisia or dystonic reactions, but it does cause increased sedation. Additionally, IV fluids, acetaminophen, or ibuprofen in conjunction with caffeine are effective. For persistent headaches, triptans can be used in the ED. In contrast, narcotics lead to significantly increased return visits and are not recommended.

#### 12. Orman, R. Swaminathan, A. Neurogenic Shock. ER Cast. (August 18, 2015). http://blog.ercast.org/spinal-shock/

This 18-minute podcast and accompanying blog post discusses the presentation, diagnosis, and evidence-based management of neurogenic shock.

##### Take-Home Points

Neurogenic shock is a form of distributive shock in patients with spinal cord injuries typically seen in patients with injury at or above T4. It is caused by a lack of sympathetic tone and presents with bradycardia and hypotension. Treatment is directed at maintaining a mean arterial pressure of over 85 mm Hg with fluid resuscitation and vasopressors (typically norepinephrine).

#### 13. Sobolewski B. Why We Do What We Do: Benzodiazepines as First Line Therapy for Status Epilepticus. (October 25, 2015). http://www.pemcincinnati.com/blog/why-we-do-what-we-do-benzodiazepines-as-first-line-therapy-for-status-epilepticus/

This evidence-based review discusses the use of benzodiazepines for status epilepticus as well as comparing lorazepam, midazolam, and diazepam in pediatrics patients older than four weeks with seizures.

##### Take-Home Points

There are several options to treat status epilepticus, which include lorazepam 0.1 mg/kg IV (maximum dose 4 mg), diazepam 0.2 mg/kg IV (maximum 8 mg), and midazolam (10 mg for > 40 kg, 5 mg for 13 – 40 kg, or 0.2 mg/kg for weight <12 kg). In head-to-head comparisons, no single benzodiazepine truly outweighs the others. The final medication recommendation depends on the patient’s access. If there is IV/intraosseous (IO) access, lorazepam is a viable option. If there is no IV/IO access, then consider IM midazolam. Rectal diazepam can be administered if IV/IO and IM access is difficult. Importantly, more than two doses of benzodiazepines increases the risk of respiratory depression.

#### 14. Butterfield M, Jeang, L. Can Giant Cell Arteritis Be Ruled Out in the ED? EM Docs. (November 14, 2015). http://www.emdocs.net/can-giant-cell-arteritis-be-ruled-out-in-the-ed/

This evidence-based blog post provides a thorough review of the utility of history, clinical exam, laboratory tests, and imaging in the evaluation of temporal arteritis.

##### Take-Home Points

Clinically ruling out temporal arteritis is difficult. Of the historical factors evaluated, the only one with significance is age as temporal arteritis is rare in patients younger than 50 years old. Even the American College of Rheumatology definition of temporal arteritis itself was designed to differentiate it from other vasculidites, and is less applicable to patients in the ED. Little evidence exists to support laboratory tests such as serum ESR or CRP to rule out temporal arteritis. Imaging studies such as MRI and ultrasound may be useful if positive, but also lack a high enough sensitivity to rule out temporal arteritis. Ultimately, the diagnostic work up for temporal arteritis is challenging, and the physician should maintain a low index of suspicion for starting steroids and arranging a temporal artery biopsy.

## CONCLUSION

The *WestJEM* Blog and Podcast Watch series serves to identify educational quality blogs and podcasts for EM clinicians through its expert panel using an objective scoring instrument. These social media resources are currently curated in the ALiEM AIR and AIR-Pro Series, originally created to address EM residency needs. These resources are herein shared and summarized to help clinicians filter the rapidly published multitude of blog posts and podcasts. Limitations include the search only includes content produced within the last 12 months from the top 50 Social Media Index sites. While these lists are by no means a comprehensive analysis of the entire Internet for these topics, this series provides a post-publication accreditation and curation of recent, online content to identify and recommend high quality, educational social media content for the EM clinician.

## Figures and Tables

**Table 1:** Approved Instructional Resources - (AIR) scoring instrument for blog and podcast content with the maximum score of 35 points.

Tier 1: BEEM rater scale	Score	Tier 2: content accuracy	Score	Tier 3: educational utility	Score	Tier 4: evidence based medicine	Score	Tier 5: referenced	Score
Assuming that the results of this article are valid, how much does this article impact on EM clinical practice?		Do you have any concerns about the accuracy of the data presented or conclusions of this article?		Are there useful educational pearls in this article for senior residents?		Does this article reflect evidence based medicine (EBM)?		Are the authors and literature clearly cited?	
Useless information	1	Yes, many concerns from many inaccuracies	1	Not required knowledge for a competent EP	1	Not EBM based, only expert opinion	1	No	1
Not really interesting, not really new, changes nothing	2		2		2		2		2
Interesting and new, but doesn’t change practice	3	Yes, a major concern about few inaccuracies	3	Yes, but there are only a few (1–2) educational pearls that will make the EP a better practitioner to know or multiple (>=3) educational pearls that are interesting or potentially useful, but rarely required or helpful for the daily practice of an EP.	3	Minimally EBM based			3
Interesting and new, has the potential to change practice	4		4				4	Yes, authors and general references are listed (but no in-line references)	4
New and important: this would probably change practice for some EPs	5	Minimal concerns over minor inaccuracies	5	Yes, there are several (>=3) educational pearls that will make the EP a better practitioner to know, or a few (1–2) every competent EP must know in their practice	5	Mostly EBM based			5
New and important: this would change practice for most EPs	6		6		6		6		6
This is a “must know” for EPs	7	No concerns over inaccuracies	7	Yes, there are multiple educational pearls that every competent EP must know in their practice	7	Yes exclusively EBM based	7	Yes, authors and in-line references are provided	7

*BEEM*, best evidence in emergency medicine; *EP,* emergency physician; *EBM,* evidence-based medicine.

**Table 2:** Blog posts and podcasts receiving an Honorable Mention on the topic of neurologic emergencies.

Title	Date	Author	Website URL
Podcast 155 – Status Epilepticus with Tom Bleck	August 13, 2015	Weingart S	http://emcrit.org/podcasts/status-epilepticus/
Treatment of Seizures in the Emergency Department: Pearls and Pitfalls.	December 17, 2015	Hernandez R, Silverberg M	http://www.emdocs.net/treatment-of-seizures-in-the-emergency-department-pearls-and-pitfalls/
Episode 73 Emergency Management of Pediatric Seizures. Emergency Medicine Cases	December 1, 2015	Richer L, Mikrogianakis A, Kilian M, Helman A	https://emergencymedicinecases.com/emergency-management-of-pediatric-seizures/
Ultrasound for Optic Nerve Sheath Diameter	December 30, 2015	Alerhand S	http://www.emdocs.net/ultrasound-for-optic-nerve-sheath-diameter/
Head Injury in Kids	March 7, 2015	Unknown	http://www.resus.com.au/blog/head-injury-in-kids/
Phenobarbital monotherapy for alcohol withdrawal: Simplicity and power	October 18, 2015	Farkas J	http://emcrit.org/pulmcrit/phenobarbital-monotherapy-for-alcohol-withdrawal-simplicity-and-power/
Concussion in Sports: Sidelines and Emergency Department Evaluation and Management	September 22, 2015	Bamman M, Williamson K, Urumov A	http://www.emdocs.net/concussion-in-sports-sideline-and-emergency-department-evaluation-and-management/
Assessing and Managing Delirium in Older Adults.	July 17, 2015	Shenvi C	http://www.aliem.com/delirium-in-older-adults/
Endovascular therapy helps in ischemic stroke, again (ESCAPE)	March 27, 2015	Rali P, Titoff I	http://pulmccm.org/main/2015/randomized-controlled-trials/endovascular-therapy-helps-in-ischemic-stroke-again-escape/
Christmas Comes Early for Endovascular Therapy in Stroke	February 12, 2015	Radecki, R	http://www.emlitofnote.com/?p=3316
Stroke Thrombolysis. Life in the Fast Lane	January 11, 2015	Nickson, C	http://lifeinthefastlane.com/ccc/stroke-thrombolysis/
Ischemic Stroke Treatment Archive	November 9, 2015	Rezaie, S	http://rebelem.com/ischemic-stroke-treatment-archive/
The Subarachnoid Enigma	May 9, 2015	Orman, R	http://blog.ercast.org/the-subarachnoid-enigma/
Pediatric Stroke: EM-Focused Highlights	August 25, 2015	Slama, R	http://www.emdocs.net/pediatric-stroke-em-focused-highlights/
Cerebral Venous Thrombosis	August 15, 2015	Nickson, C	http://lifeinthefastlane.com/ccc/cerebral-venous-thrombosis/
Episode 17 Part 1: Emergency Stroke Controversies	January, 2015	Himmel W, Selchen D, Chartier L, Helman A	https://emergencymedicinecases.com/episode-17-part-1-emergency-stroke-controversies/
SGEM#137: A Foggy Day – Endovascular Treatment for Acute Ischemic Stroke	November 21, 2015	Spiegel, R	http://thesgem.com/2015/11/sgem137-a-foggy-day-endovascular-treatment-for-acute-ischemic-stroke/
The Approach to the Dizzy Patient	November 17, 2015	Hill J, McKean J, Knight B	http://www.tamingthesru.com/blog/bread-and-butter/dizziness?rq=stroke
Stroke and TIA: Pearls and Pitfalls	May 29, 2015	Ferguson W, Crane D, Lo A	http://www.emdocs.net/stroke-and-tia-pearls-and-pitfalls/
Tissue, Not Time, for Stroke	September 18, 2015	Radecki, R	http://www.emlitofnote.com/?p=3229

*TIA,* transient ischemic attack
